# Full-field electroretinogram (ffERG) over 48 months correlates with baseline retinal dysfunction in Vogt-Koyanagi-Harada disease: a longitudinal study

**DOI:** 10.1007/s10633-025-10080-9

**Published:** 2026-01-30

**Authors:** Fernanda Maria Silveira Souto, Ruy Felippe Brito Gonçalves Missaka, Marcelo Mendes Lavezzo, Viviane Mayumi Sakata, Maria Kiyoko Oyamada, Joyce Hisae Yamamoto, Brazilian Vogt-Koyanagi-Harada disease Study Group, Fernanda Maria Silveira Souto, Ruy Felippe Brito Gonçalves Missaka, Marcelo Mendes Lavezzo, Viviane Mayumi Sakata, Maria Kiyoko Oyamada, Joyce Hisae Yamamoto, Priscilla Figueiredo Campos da Nóbrega, Camillo Carneiro Gusmão, Emmett Cunningham, Carlos Eduardo Hirata

**Affiliations:** 1 Department of Ophthalmology, Hospital das Clinicas, HCFMUSP, Faculdade de Medicina FMUSP, Universidade de Sao Paulo, Sao Paulo, SP Brazil; 2 Department of Ophthalmology, Faculdade de Medicina FMUSP, Hospital das Clinicas, HCFMUSP, Universidade de Sao Paulo, LIM 33, Sao Paulo, SP Brazil; 3 West Coast Medical Group, San Francisco, CA USA; 4 Department of Ophthalmology, California Pacifical Medical Center, San Francisco, CA USA; 5 Department of Ophthalmology, Stanford University School of Medicine, Stanford, CA USA; 6 The Francis I Proctor Foundation, UCSF School of Medicine, San Francisco, USA

**Keywords:** Vogt-Koyanagi-Harada disease, Inflammation, Electrophysiology, Uveitis, Therapeutics

## Abstract

**Objective:**

To evaluate full-field electroretinogram (ffERG) parameters and evaluate their clinical associations in patients with acute Vogt-Koyanagi-Harada (VKH) disease over a 4-year follow-up.

**Methods and analysis:**

This retrospective cohort study included 21 patients with acute VKH disease followed for 48 months after initiation of systemic corticosteroid therapy, with or without adjunctive immunosuppression. ffERG was performed at 1, 6, 12, and 48 months (M) post-treatment. At M48, eyes were classified into two groups: Group 1 (normal ffERG) and Group 2 (subnormal ffERG), based on whether any ffERG parameters fell below the 5th percentile of age-matched healthy controls. Main outcomes included longitudinal ffERG changes, clinical associations, and the 6-month recovery ratios relative to baseline (M1).

**Results:**

All ffERG parameters improved significantly from baseline to M6 (p < 0.001) and to M12 (p < 0.001) and stabilized thereafter. Group 2 exhibited consistently reduced ffERG amplitudes compared to Group 1 throughout follow-up (p < 0.01 to p < 0.001), despite similar recovery trends. Recovery ratios at M6 ranged from 27%–78% in Group 1 and 26%–175% in Group 2; however, Group 2 remained below normal levels at M48. Sunset glow fundus (SGF) at M12 was significantly more frequent in Group 2 (60.7%) than in Group 1 (21.4%, p = 0.025).

**Conclusions:**

Retinal function improved during the first year and stabilized thereafter, irrespective of treatment type. Persistent subnormal ffERG at 48 months reflected a poorer baseline function and was associated with the development of SGF.

**Supplementary Information:**

The online version contains supplementary material available at 10.1007/s10633-025-10080-9.

## Introduction

Vogt-Koyanagi-Harada (VKH) disease is a systemic autoimmune disorder targeting pigmented tissues—including the choroid, meninges, and skin—in genetically susceptible individuals. [1] VKH disease accounts for 2 to 13% of uveitis cases seen in tertiary centers in Brazil and is a leading cause of non-infectious uveitis, predominantly affecting young women [1, 2]. The acute onset of VKH disease is marked by bilateral panuveitis, optic disc hyperemia and/or edema, and serous retinal detachment, with distinctive findings on multimodal imaging. [1] Prompt initiation of high-dose systemic corticosteroids followed by gradual tapering of oral prednisone typically results in favorable visual outcomes; one year after disease onset, 83% to 93% of patients achieve visual acuity of 20/25 or better. [3, 4] However, subclinical choroidal inflammation often persists, as revealed by posterior segment multimodal imaging, contributing to a high incidence of structural complications such as sunset glow fundus (SGF), subretinal fibrosis, chorioretinal atrophy, and choroidal neovascular membrane. Consequently, uveitis specialists advocate for the early initiation of systemic non-steroidal immunosuppressive therapy (IMT) to reduce long-term ocular complications and limit prolonged corticosteroid use. [5–8]

Recently, our group published a 24-month longitudinal study analyzing full-field electroretinograms (ffERGs) in 12 patients with acute VKH disease. [3] All patients were treated with high-dose systemic corticosteroids, with late initiation of non-steroidal IMT in refractory cases. Although ffERG parameters initially improved in all eyes, 29% exhibited deterioration between months 12 and 24. By the 24 months, 62% of eyes exhibited subnormal ERG responses. No significant association was found between retinal function decline and the treatment regimen; however, increased choroidal thickness, either shortly after treatment initiation or intermittently during follow-up, correlated with worsening ffERG parameters. [3] A subsequent cohort of 11 patients with acute VKH disease receiving early azathioprine addition showed similar functional and disease courses. [9] These findings suggest that visual function, as measured by ffERG, may primarily reflect disease severity at onset.

Therefore, this retrospective cohort study aimed to evaluate the four-year longitudinal trends in ffERG parameters and their association with clinical findings in patients following acute onset of VKH disease.

## Material and methods

### Study design and sample

This retrospective interventional cohort study included 21 patients (42 eyes) diagnosed with VKH disease [10], and followed at the Uveitis Service, Department of Ophthalmology, Hospital das Clinicas HCFMUSP, Faculdade de Medicina, Universidade de São Paulo, Brazil, between June 1, 2011, and December 31, 2021. All patients participated in the longitudinal VKH disease study (clinical trials NCT03811366 and NCT03399175) and were followed from acute disease onset through systematic multimodal and functional evaluations, according to predefined treatment protocols. Exclusion criteria were refractive errors greater than 5 diopters, inadequate mydriasis, or media opacities. This study was approved by the local Institutional Ethics Committee (CapPesq 0496–11). Written informed consent was obtained from all participants, and all procedures adhered to the tenets of the Declaration of Helsinki.

Initial treatment comprised a three-day intravenous methylprednisolone pulse therapy (1 g/day), followed by oral prednisone (1 mg/kg/day) with a gradual tapering over at least nine months. IMT—including azathioprine, mycophenolate mofetil, and/or cyclosporine A- was added as a corticosteroid-sparing treatment and/or for refractory disease or prednisone intolerance. Systemic treatment escalation during follow-up was based on clinical relapses and/or worsening of ffERG parameters. Worsening of the ffERG was defined as a reduction of ≥ 30% in any dark adapted ERG parameter, confirmed on two consecutive examinations [3]. Isolated persistence or worsening of subclinical inflammation did not prompt treatment escalation.

The longitudinal VKH disease study protocol included clinical and posterior segment imaging examinations—fundus color photography, indocyanine green angiography (ICGA), fluorescein angiography (FA), and enhanced depth imaging optical coherence tomography (EDI-OCT)—performed at baseline, 1, 3 and 6 months, and every three months thereafter. Clinical data were extracted from medical records.

Clinical relapses after acute onset were defined as anterior and/or posterior uveitis flares and/or macular oedema confirmed by FA and/or OCT. Fundus photography was performed using the Topcon® TRC-50DX (Tokyo, Japan). SGF development was classified as present or absent. FA, ICGA, and EDI- OCT were performed using the Spectralis® HRA + OCT (Heidelberg Engineering, Germany). FA and ICGA reading charts were adapted from Tugal-Tutkun et al.[11] Dark dots were scored semi-quantitatively over the posterior pole and quadrants, categorized as sparse/faint, or numerous/pronounced, with total scores ranging from 0 to 8.

### Electroretinogram evaluation

Ff-ERGs were recorded using the RETI-port system (Roland Consult, Germany) following the Standards of the International Society for Clinical Electrophysiology of Vision.[12] ERG-jet electrodes (Universe SA, La Chaux-de-Fonds, Switzerland) were used for all recordings. Dark adapted (DA) ERGs were recorded after 20 min of dark adaptation, and light adapted (LA) ERGs after 10 min of light adaptation. The standardized recording sequence included the DA 0.1 (rod system) ERG, the DA 3.0 (cone-rod) ERG, the DA oscillatory potentials, the LA 3.0 (cone system) ERG, and the 30-Hz flicker ERG (cone). ffERG examinations were performed at presentation, 30 days post-treatment, and then at six-month intervals. For analysis, data from the first (M1), sixth (M6), twelfth (M12), and forty-eighth (M48) months were used. Given the limited feasibility and reliability of ffERG recordings at presentation, primarily due to acute serous retinal detachment and emergency-care logistical constraints, M1 was adopted as the earliest standardized and clinically interpretable baseline for longitudinal analysis. For the purpose of this study, M1 was considered the functional “baseline” state after initial treatment. At M48, eyes were classified into two groups based on amplitude parameters: Group 1 (normal ffERG) and Group 2 (subnormal ffERG). A subnormal ffERG was defined as having at least one parameter below the 5th percentile of age- and gender-matched healthy controls. Normal value limits for each ffERG parameter were established using 95% confidence intervals. Amplitude of *a-* and *b-* waves from eight averaged stimulus responses were analyzed and compared with a normative database.

### Outcomes

The main outcome assessed was the longitudinal ffERG changes observed in Groups 1 and 2 throughout the 48-month follow-up.

### Data analysis

Descriptive statistics were summarized using medians, ranges, means, and standard deviation (± SD). Best corrected visual acuity (BCVA), measured with a Snellen chart, was converted to logarithm of the minimal angle of resolution (LogMAR) units. For each eye and each ffERG parameter, a recovery ratio was computed by dividing the amplitude measured at M6 by the corresponding amplitude at M1. This yielded a proportional measure of functional change over time, where a ratio > 1 indicated improvement relative to baseline, a ratio = 1 indicated stability, and a ratio < 1 indicated functional decline. Recovery ratios were summarized using median and range for Groups 1 and 2.

For binary ocular outcomes, generalized estimation equations (GEE) were applied with an identity link function and an interchangeable correlation matrix to account for inter-eye correlations. Longitudinal continuous ffERG parameters were analyzed using GEE with a normal distribution and identity link function, including group (Group 1 vs Group 2) and timepoint (M1, M6, M12, M48) as fixed factors. An autoregressive (AR-1) correlation structure was specified to account for repeated measurements over time and correlation between eyes within the same patient. Pairwise comparisons between groups at each timepoint were derived from these models, with p < 0.05 considered statistically significant. Additional GEE models with binomial distribution and logit link, or with Poisson distribution and identity link, were applied for binary or count outcomes as appropriate. Because the number of pairwise comparisons generated a large amount of numerical information, all detailed p-values for longitudinal and intergroup analyses were organized into the supplementary table to improve readability. *P*-values < 0.05 were considered significant. Statistical analyses were conducted using the SPSS version 22.0 (SPSS Inc., Chicago, Illinois, USA).

## Results

A total of 21 patients (42 eyes) with acute VKH disease, with a mean age at diagnosis of 32.8 ± 12.3 years, were included; 19 (90.5%) were assigned female at birth. The mean interval between symptoms onset and diagnosis was 23.9 ± 16.6 days. Mean visual acuity at presentation was 1.5 ± 0.9 logMAR, improving to 0.4 ± 0.7 at M1 and 0.1 ± 0.2 logMAR at M48. At presentation, serous retinal detachment was observed in 32 eyes (76.2%) and bacillary layer detachment in 17 eyes (40.5%). The mean AF and ICGA scores at presentation were 6.1 ± 3.0 and 11.7 ± 2.4, respectively. At M12, fibrosis was observed in 15 eyes (35.7%) and SGF in 20 eyes (47.6%). The mean number of anterior uveitis relapses over 48 months was 0.6 ± 1.3. All patients received initial methylprednisolone pulse therapy. During follow-up, 15 patients (71.4%) received additional non-corticosteroid IMT, while 6 patients (28.6%) remained on corticosteroids alone.

At M48, 14 eyes (33.3%) were classified in Group 1 (normal ffERG) and 28 eyes (66.7%) in Group 2 (subnormal ffERG). Clinical characteristics by group are summarized in Table 1.

**Table 1:** Clinical characteristics of 21 patients (42 eyes) with non-acute Vogt-Koyanagi-Harada disease, classified into Groups 1 and 2 based on full-field electroretinogram parameters at 48 months

Description	All patients (n = 42 eyes)	Group 1 (n = 14 eyes)	Group 2 (n = 28 eyes)	p*
*Clinical data*				
Age at diagnosis, years	32.8 ± 12.3 32 (15–67)	32.9 ± 6.4 32.5 (19–42)	32.8 ± 14.5 29 (15–67)	0.989
Time to start treatment, days	23.9 ± 16.6 21 (3–67)	18.3 ± 11.3 17.5 (3–44)	26.7 ± 18.2 22 (7–67)	0.220
Pleocytosis	149.9 ± 149.8 119 (0–590)	93.6 ± 84.3 102 (0–198)	176.9 ± 167.4 130 (0–590)	0.220
BCVA, logMAR				
At M0	1.5 ± 0.9 2 (0–3)	1.4 ± 0.8 2 (0–2)	1.6 ± 1.0 2 (0–3)	0.122
At M1	0.4 ± 0.7 0.2 (0–3)	0.3 ± 0.3 0.25 (0–1)	0.5 ± 0.9 0.17 (0–3)	0.169
At M48	0.1 ± 0.2 0 (0–1)	0.1 ± 0.1 0 (0–0.5)	0.1 ± 0.2 0 (0–1)	0.269
*Disease activity*				
Fluorescein angiography score				
At M0	6.1 ± 3.0 6 (0–10)	3.6 ± 2.7 5 (0–10)	6.9 ± 1.9 7 (2–9)	0.217
At M1	2.9 ± 2.1 3 (0–7)	1.6 ± 1.6 2.5 (0–5)	3.9 ± 1.9 3 (0–7)	0.178
Indocyanine green angiography score				
At M0	11.7 ± 2.4 12 (7–18)	11.6 ± 2.0 12 (7–15)	11.8 ± 2.7 11 (8–18)	0.575
At M1	10.7 ± 1.5 11 (6.5–14)	10.5 ± 1.6 11 (6.5–14)	10.8 ± 1.4 11 (8.5–14)	0.866
Choroidal thickness at M1, μm	569.7 ± 158.9 541 (300–911)	528.5 ± 134.1 533 (300–787)	590.3 ± 168.4 549 (303–911)	0.439
Number of anterior uveitis relapse episodes up to M48				
	0.6 ± 1.3 0 (0–5)	0.6 ± 1.2 0 (0–4)	0.6 ± 1.4 0 (0–5)	0.776
*Fundus appearance*, n eyes (%)				
Fibrosis at M12	15 (35.7)	3 (21.4)	12 (42.9)	0.323
Chorioretinal atrophy at M12	12 (28.6)	3 (21.4)	9 (32.1)	0.594
Peripapillary atrophy at M12	14 (33.3)	5 (35.7)	9 (32.1)	0.626
Sunset glow fundus at M12	20 (47.6)	3 (21.4)	17 (60.7)	**0.025**
Choroidal neovascular membrane	8 (19.0)	2 (14.3)	6 (21.4)	0.664
*Treatment start,* n eyes (%)				
Corticosteroid				0.484
< 14 days	16 (38.1)	7 (50.0)	9 (32.1)	
14- 30 days	14 (33.3)	5 (35.7)	9 (32.1)	
≥ 30 days	12 (28.6)	2 (14.3)	10 (35.7)	
Non-steroidal immunosuppressive therapy				0.396
No use	12 (28.6)	4 (28.6)	8 (28.6)	
< 30 days	4 (9.5)	3 (21.4)	1 (3.6)	
≥ 30 days	26 (61.9)	7 (50.0)	19 (67.9)	

Data are presented as mean ± standard deviation and median (range) for clinical and disease activity variables

Eyes were classified as normal (Group 1) or subnormal (Group 2) based on ffERG results at 48 months. A subnormal ffERG recording was defined as the presence of at least one parameter below the fifth percentile of age- and gender-matched healthy controls

*Generalized estimated equations with normal distribution and logarithmic link function, supposing an interchangeable correlation matrix between the eyes: p-value < 0.05 indicated a statistical significance

IMT: immunosuppressive therapy M0: month 0; M1: month 1; M12: month 12; M48: month 48; SD: standard deviation; BCVA: best corrected visual acuity

The groups 1 and 2 showed no significant differences in age at diagnosis (p = 0.989) or interval to treatment initiation (18.3 ± 11.3 vs 26.7 ± 18.3 days, p = 0.220) or in any of the clinical measures:. While BCVA was slightly worse in Group 2 at presentation (1.6 ± 1.0 vs 1.4 ± 0.8 logMAR, p = 0.122) and at M1 (0.5 ± 0.9 vs 0.3 ± 0.3 logMAR, p = 0.169), differences were not statistically significant. FA scores were higher in Group 2 at both presentation (3.6 ± 2.7 vs 6.9 ± 1.9, p = 0.217) and M1 (1.6 ±1.6 vs 3.9 ± 1.9, p=0.178)), but without significance. At M12, fibrosis (42.9% vs 21.4%, p = 0.323), chorioretinal atrophy (32.1% vs 21.4%, p = 0.594), and choroidal neovascular membrane (21.4% vs 14.3%, p = 0.664) were more frequent in Group 2, though only SGF was significantly more common (60.7% vs 21.5%, p = 0.025).

A late corticosteroid initiation (≥ 30 days from disease onset) was more frequent in Group 2 (35.7% vs 14.3%, p = 0.484), whereas early IMT initiation was more common in Group 1 (21.5% vs 3.6%, p = 0.396), though neither reached statistical significance.

Concerning ffERG results, Table 2 summarizes the median and range of dark and light adapted ffERG amplitude parameters across four timepoints (M1, M6, M12, M48), reported for all 42 eyes collectively and stratified by Group 1 (normal ffERG at M48) and Group 2 (subnormal ffERG at M48).

**Table 2:** FfERG results (median and range) of 21 patients (42 eyes) with Vogt-Koyanagi-Harada disease at 1, 6, 12 and 48 months after acute disease onset, classified into Groups 1 and 2 based on ffERG parameters at 48 months

Full-field ERG parameters		All patients (n = 42 eyes)	Group 1 (n = 14 eyes)	Group 2 (n = 28 eyes)	Controls
*Dark adapted 0.1* *b* amplitude (μV)	M1	85.0 (0.0–343.0)	149.0 (34.8–343.0)	63.8 (6.7–202.0)	211.0 * (114–366)
	M6	184.5 (46.3–460.0)	244.0 (176.0–382.0)	163.0 (46.3–460.0)	
	M12	201.0 (104.0–352.0)	220.1 (164–352)	184.5 (104.0–300.0)	
	M48	190.3 (96.6–372.0)	272.0 (198.5–372.0)	154.5 (96.6–360.0)	
Dark adapted 3.0 *a* amplitude (μV)	M1	153.5 (39.4–419.0)	217.0 (72.2–419.0)	144.0 (39.4–310.0)	300.0 (211.0- 440.0)
	M6	230.5 (29.4–319.3)	264.0 (224.0- 319.3)	203.0 (29.4- 309.0)	
	M12	215.0 (89.4- 394.0)	269.5 (186.0- 394.0)	199.5 (89.4- 268.0)	
	M48	213.2 (77.3–396.0)	310.5 (243.0- 396.0)	187.0 (77.3- 267.0)	
*Dark adapted 3.0* *b* amplitude (μV)	M1	315.0 (73.5- 798.0)	451.0 (244.0- 798.0)	265.0 (73.5–536.0)	510.0 * (348.0- 789.0)
	M6	486.5 (89.2- 731.0)	546.0 (470.0- 731.0)	441.0 (89.2- 677.0)	
	M12	495.0 (313.0- 750.0)	568.6 (481.3–750.0)	435.5 (313.0- 658.0)	
	M48	446.6 (293.1- 776.5)	575.7 (456.8- 776.5)	410.0 (293.1- 646.0)	
Oscillatory potential (μV)	M1	58.5 (0.0- 302.1)	72.9 (23.5- 302.1)	49.7 (13.4- 180.7)	121.0* (92.0–228.0)
	M6	100.8 (27.6- 286.9)	131.9 (58.3–286.9)	91.7 (27.6–212.5)	
	M12	110.0 (48.5- 297.0)	161.3 (59.1–297.0)	85.3 (48.5- 205.7)	
	M48	117.5 (37.5- 253.6)	169.6 (112.0- 253.6)	106.8 (37.5- 189.5)	
*Light adapted 3.0* *a* amplitude (μV)	M1	32.8 (14.6- 132.0)	36.9 (19.6- 67.7)	31.3 (14.6–132.0)	50.0 * (34.0–89.0)
	M6	40.1 (18.1- 57.9)	42.7 (34.9- 57.9)	34.3 (18.1- 57.6)	
	M12	39.2 (16.7- 63.3)	42.8 (26.7- 63.3)	33.4 (16.7- 49.1)	
	M48	38.9 (23.4- 76.6)	48.6 (35.3- 76.6)	37.7 (23.4- 52.1)	
*Light adapted* *b* amplitude (μV)	M1	125.5 (19.7- 315.0)	110.0 (81.2–280.0)	126.0 (19.7- 315.0)	227.0 (106.0- 367.0)
	M6	168.0 (77.9- 323.0)	178.0 (158.2- 337.4)	157.0 (81.8- 326.0)	
	M12	168.8 (77.9- 323.0)	210.0 (140.0- 323.0)	157.9 (77.9- 300.0)	
	M48	174.4 (103.0- 397.0)	201.0 (157.3- 397.0)	159.0 (102.0- 268.9)	
Flicker 30 Hz (peak to peak μV)	M1	32.8 (0.0- 102.0)	45.4 (14.0- 77.9)	31.8 (9.0- 102.0)	68.0 * (42.0- 122.0)
	M6	49.2 (20.2- 94.2)	54.0 (35.6- 88.8)	45.1 (20.2- 94.2)	
	M12	44.6 (21.8- 86.6)	56.5 (41.0- 81.4)	42.3 (21.8- 86.6)	
	M48	49.9 (33.7- 106.0)	57.6 (51.0- 106.0)	44.2 (33.7- 77.3)	

Eyes were classified as normal (group 1), and subnormal (group 2) based on ffERG results at 48 months. A subnormal ffERG recording was defined as the presence of at least one parameter below the fifth percentile of healthy age- and gender-matched controls. * Detailed p-values for all within-group and between-group comparisons across timepoints were moved to Supplementary Table for clarity. Statistical analysis was performed using generalized estimating equations (normal distribution, identity link, AR-1 correlation structure), with p < 0.05 considered significant

At M1, eyes in Group 1 demonstrated significantly reduced amplitudes compared to age-matched healthy controls in both DA ERG parameters— *DA 0.1 b*-wave amplitude (p = 0.033), *DA 3.0* *a*-wave amplitude (p < 0.001), and *DA 3.0* *b*-wave amplitude (p = 0.022)—and in LA parameters—*LA 3.0* *b*-wave amplitude (p = 0.011) and LA flicker response (p = 0.004).

**Fig. 1:**
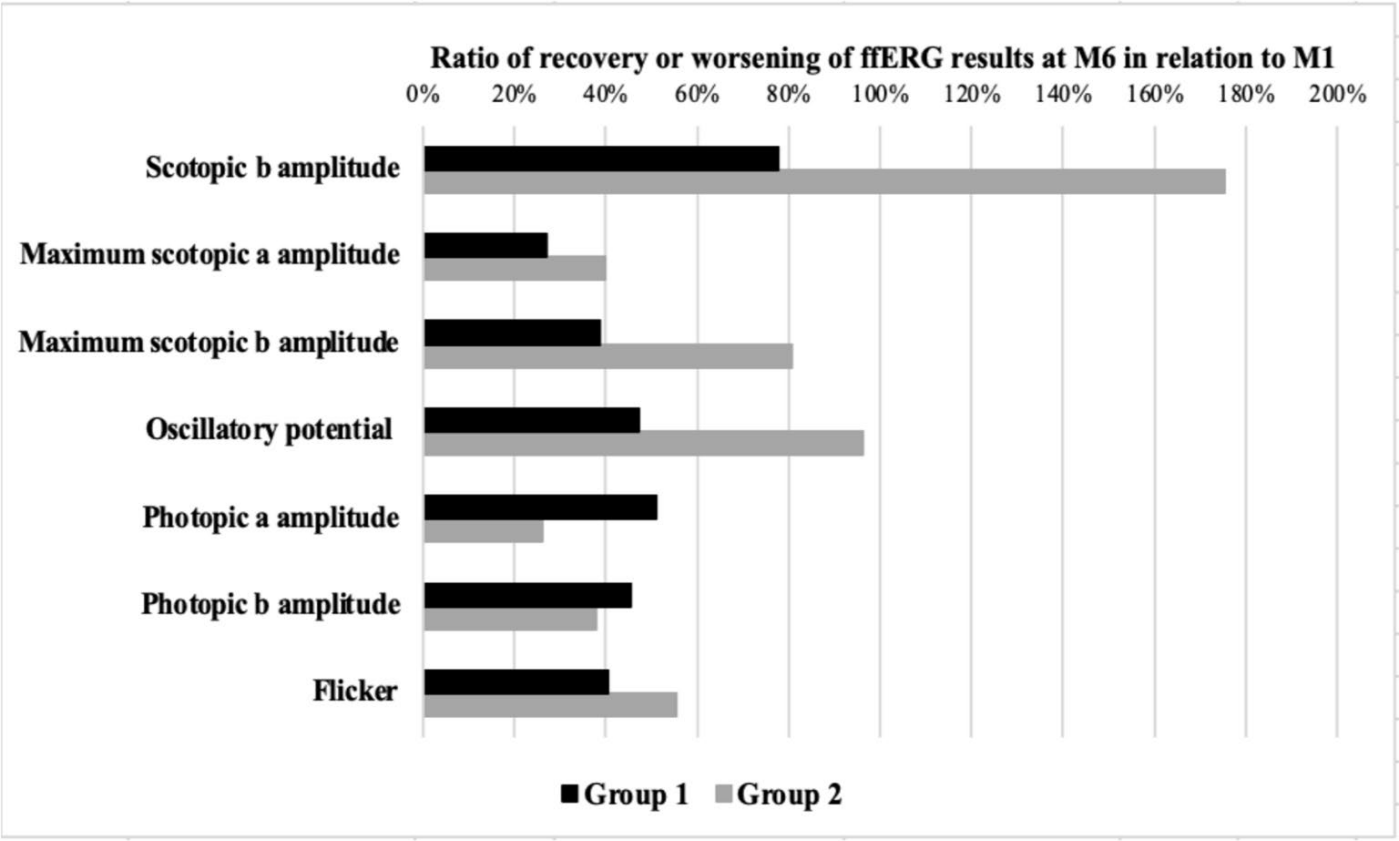
Median ratio of recovery or worsening of ffERG results in Groups 1 and 2 at M6 relative to M1

The Fig. 1 shows the recovery ratios of ffERG parameters at M6 relative to M1. In Group 1, a median recovery was observed across all parameters: *DA 0.1* *b*-wave, 77.8% (range: -2.1 to 477.6%); *DA 3.0* *a*-wave, 27.1% (-15.8 to 214.3%); *DA 3.0* *b*-wave, 38.8% (-10.5 to 123.1%); oscillatory potentials, 47.2% (-19.7 to 482.9); *LA 3.0 a*-wave, 51.1% (-6.9 to 155.6); *LA 3.0* *b*-wave, 45.6% (-15.2 to 104.4); and LA flicker response, 40.6% (3.0 to 191.4).

Despite recovery, some parameters in Group 1 remained significantly lower than controls at M6, including *DA 3.0* *a*-wave amplitude (p = 0.002), *LA 3.0* *b*-wave amplitude (p = 0.038), and flicker response (p = 0.046). At M12, the flicker response was still reduced (p = 0.015), and by M48, *DA 3.0 a*-wave amplitude was again significantly lower than that of controls (p = 0.001), despite prior normalization.

In Group 2, all ffERG parameters were significantly reduced at M1 compared to controls (p < 0.001). A median recovery was also observed across all parameters (Fig. 1): *DA 0.1* *b*-wave, 175.4% (range: -0.5 to 2,031.1%); *DA 3.0* *a*-wave, 39.9% (-70.5 to 127.6%); *DA 3.0* *b*-wave, 80.7% (-64.0 to 229.2); oscillatory potentials, 96.1% (-44.4 to 402.8); *LA 3.0* *a*-wave, 26.0% (-43 to 121.9); *LA 3.0* *b*-wave, 37.8% (-40.3 to 509.1); and flicker response, 55.5% (-9.7 to 234.4).

However, except for the *DA 0.1* *b-*wave amplitude, all parameters remained significantly reduced at M6 compared to those of controls (p < 0.001). By M12, the*DA 0.1* *b-*wave amplitude was again reduced (p = 0.01), while all other parameters remained significantly lower than those of controls (p < 0.001). At M48, only *DA 3.0* *b-*wave amplitude (p = 0.304) and oscillatory potentials (p = 0.133) normalized; all others remained significantly reduced (p = 0.029 to < 0.001).

**Fig. 2:**
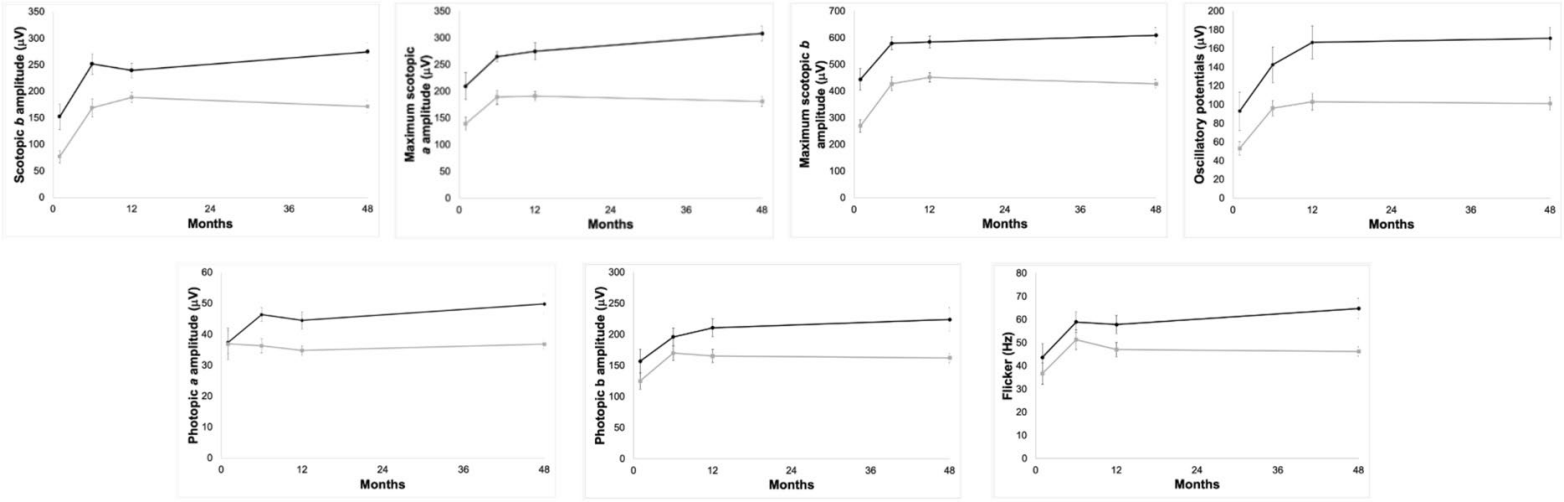
Graphical presentation of ffERG results (mean and standard deviation) at four timepoints for DA 0.1 *b*-wave amplitude, DA 3.0 *b*-wave amplitude, oscillatory potentials and flicker response of Groups 1 and 2

As shown in Fig. 2**,** curves for DA 0.1 *b-*wave, maximal DA 3.0 *b-*wave, oscillatory potentials, and flicker ERg amplitude revealed similar trends in both groups: a significant improvement at M6 compared to M1 (p < 0.001), followed by stabilization through M12 and M48. However, values were consistently lower in Group 2 at all timepoints (e.g., scotopic *b-*wave at M1, p < 0.001).

DA3.0 *a-*wave amplitude differed significantly between groups at all timepoints (p = 0.03 to p < 0.001), with both groups showing initial gains at M6 and M12. At M48, however, only Group 1 sustained significant improvement from M1 (p < 0.001), while Group 2 did not (p = 0.066), suggesting late functional decline.

For LA 3.0 *a-*wave amplitude, overall intergroup differences were significant (p = 0.01), though no individual timepoint showed statistical significance.LA 3.0 *b-*wave amplitude improved similarly in both groups between M1 and M6 and remained stable at M12 and M48 (p = 0.002 to p < 0.001), but a significant intergroup difference emerged only at M48 (p < 0.001).

During the 48-month follow-up, ffERG worsening episodes occurred in both groups: 8/14 eyes (57%) in Group 1 and 21/28 eyes (75%) in Group 2 (p = 0.298). Notably, 10 eyes in Group 2 experienced multiple episodes. Each episode of confirmed ffERG worsening led to an escalation in immunosuppressive treatment.

**Fig. 3:**
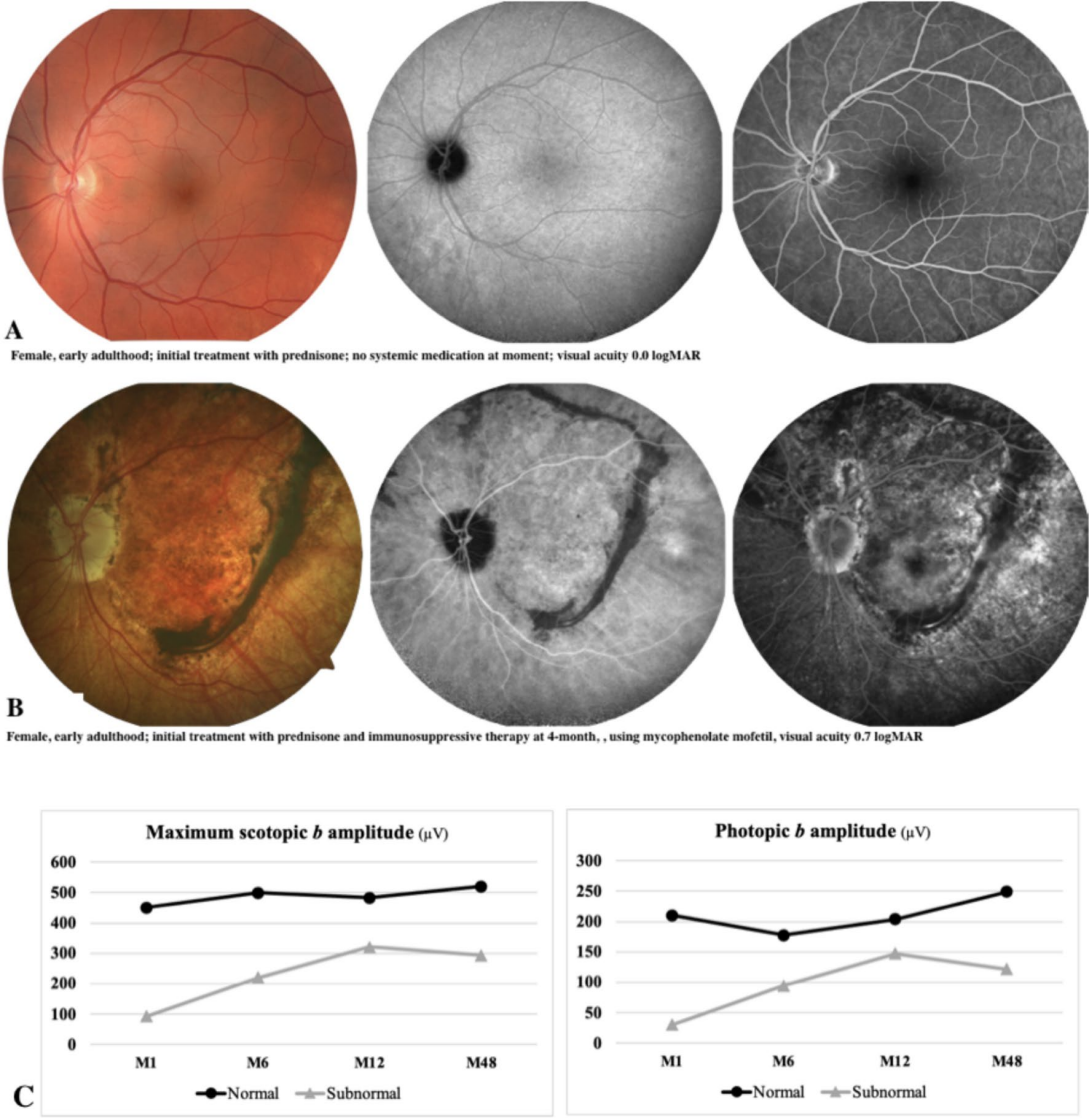
Representative examinations of two patients with Vogt-Koyanagi-Harada disease at the 48-month follow-up evaluation. **A** Fundus color photography (left), late-phase indocyanine green angiography (center), late-phase fluorescein angiography (right) of a patient with normal ffERG (Group 1) at 48 months. **B** Same imaging modalities in a patient with subnormal ffERG (Group 2) at 48 months. **C** Corresponding ffERG behavior showing changes in DA 3.0 *b*-wave and *LA 3.0 b*-wave amplitudes over time in these two patients

Representative multimodal imaging and ffERG data from two patients with VKH disease at M48 are shown in Fig. 3. A supplementary figure with the representative ffERG waveforms is provided.

## Discussion

In this retrospective study, we evaluated the longitudinal behavior of retinal function—measured by ffERG- in 21 patients with VKH disease followed for four years after acute onset. Although VKH disease primarily targets the choroidal stroma, it can severely compromise the outer retina in the acute phase. [13] Serous retinal detachment, including bacillary layer detachment, along with findings from multimodal imaging and functional tests, supports the notion of early outer retinal involvement. [3, 14–16] Despite this, VKH disease generally has a favorable BCVA prognosis when promptly and adequately treated during the early acute phase. [3, 4] However, even in patients with good BCVA, late fundus complications—such as SGF, subretinal fibrosis, choroidal neovascularization, and persistent deficits in retinal sensitivity or color vision—suggest ongoing structural and functional retinal impairment [17–19].

In our cohort, ffERG parameters were markedly impaired at disease onset in all patients; however, a rapid initial improvement was observed within the first six months in all eyes. This indicates that ffERG reliably reflects VKH disease-related retinal dysfunction across both acute and chronic phases. [3, 20–22] Early case reports by Jacobson et al.[20] and Godel et al.[22] similarly described marked ffERG amplitude reductions during acute retinal detachment with gradual recovery over time, though long-term deficits persisted. Two prospective studies have evaluated ffERG behavior in patients with VKH disease. [3, 21] Yuan et al. [21]followed 42 patients and reported recovery of all ffERG parameters—except the *DA 0.1* *a*-wave- by 12 months after corticosteroid and cyclosporine therapy. [21] In contrast, Sakata et al. [3]found that only 37.5% of 24 eyes normalized their ffERG by 24 months. Methodological differences likely contributed to these contrasting findings. Yuan’s study [21]had high loss of follow-up (only 42.8% of eyes completed 12-month testing), while Sakata’s study [3]maintained full follow-up and every ffERG worsening episodes was confirmed by a second examination.

In our study, all 21 patients underwent systematic multimodal imaging and ffERG testing at fixed intervals per protocol. Eyes were classified by their ffERG results at M48 into Group 1 (normal ffERG) and Group 2 (subnormal ffERG). All ffERG parameters—initially severely affected—improved significantly between 6 and 12 months (p < 0.001), after which they stabilized, indicating early widespread retinal dysfunction with partial recovery. A more compromised ffERG at baseline and the presence of SGF at 12 months were both associated with persisted dysfunction at 48 months.

Compared to healthy controls, several ffERG parameters in Group 1 were significantly reduced. At M1, all parameters were impaired except for oscillatory potential and photopic *a*-wave amplitude. At M6, significant reductions persisted in DA 3.0 *b-*wave amplitude, photopic *b-*wave amplitude and flicker response. By M12, only the flicker response remained reduced, and at M48, the DA 3.0 *a-*wave—which had recovered at M12- showed a decline again.

Group 2 eyes had all ffERG parameters significantly reduced at M1 compared to controls, and most remained subnormal at M48, except for DA 0.1 *b-*wave and oscillatory potential.

These findings suggest that a severe baseline dysfunction—even when followed by high relative recovery- predicts long-term impairment. In Group 2, treatment intensification, guided by clinical relapses and/or ffERG worsening, led to more frequent escalation of IMT, which may have prevented further functional deterioration. Nevertheless, this aggressive management was insufficient to fully restore retinal function, highlighting the prognostic significance of early, severe dysfunction.

An analysis of recovery ratios at M6 relative to M1 illustrated that Group 1 had modest gains (e.g., 27.1% for DA 3.0 a-wave, 77.8% for scotopic b-wave), whereas Group 2 had broader gain variability (e.g., 26% for LA 3.0 a-wave, 175.4% for the DA 0.1 b-wave). Despite these gains, ffERG remained subnormal in Group 2. We should note that due to markedly reduced M1 amplitudes in Group 2, some recovery ratios exceeded 1000%. These values should not be interpreted as supranormal recovery, but rather as improvements from severely depressed post-acute function. Therefore, the recovery ratio reflects initial severity rather than true normalization, and absolute changes from M1 to M6 should also be considered when interpreting the magnitude of functional improvement.

Interestingly, recovery of dark adapted parameters—except for the DA 3.0 *a-*wave—was more pronounced than light adapted recovery in both groups. This contrasts with earlier studies and warrants future investigation. [20–22] Notably, Jacobson et al. [20] described persistent loss of dark adapted ERG components despite recovery of light adapted function, highlighting a rod-predominant vulnerability that differs from the relative photopic recovery observed in our cohort.

Contrary to expectations, we found no significant association between treatment delay and baseline ffERG severity. However, the small number of patients in each group substantially may limit the statistical power of these analyses. Consequently, the possibility of a type II error cannot be excluded, and the non-significant findings should be interpreted with caution. These exploratory results require confirmation in studies with larger cohorts.

Although many studies have investigated ffERG findings in birdshot retinochoroiditis (BRC), the results and clinical implications differ significantly from those in VKH disease. [13, 23, 24] Hirose et al. [23]reported stage-dependent ffERG changes in BRC, with a characteristic “negative type” ERG—marked reduced dark adapted *b*-wave, followed by light adapted *b*-wave and flicker abnormalities—suggesting a more diffuse and severe involvement of the neural retinal layers than the receptor-retinal pigment epithelium-choroid complex. Zachs et al. [25] showed that strong dark adapted amplitudes and 30 Hz flicker implicit times are useful markers of disease activity and treatment guidance. More recently, Wang et al.[26] found oscillatory potentials offer higher sensitivity and specificity for diagnosing and monitoring BRC than previously used ffERG parameters. Retinal function in BRC progressively deteriorates over time, despite preserved BCVA and apparent disease control. [27, 28] Thus, ffERG in BRC serves as a valuable, objective tool to assess function progression, evaluate treatment, guide timely adjustments in therapy [25–28].

The main limitation of this study is its modest sample size. Despite this, statistically significant findings were observed using this  small number of patients in each group, which  substantially limited the statistical power of the analyses. Consequently, the possibility of a type II error cannot be excluded, and the non-significant findings should be interpreted with caution. Notably, this is the first study to assess retinal function with ffERG in VKH disease over a 48-month period. Our longitudinal data provide valuable insights into the long-term functional outcomes in non-acute VKH disease. Further studies are needed to clarify the relationship between ffERG changes, structural alterations, and treatment response, and these exploratory results require confirmation in studies with larger cohorts.

In conclusion, this 48-month follow-up identified two distinct VKH disease groups based on ffERG outcomes: one with normalized function and another with persistent dysfunction despite initial high recovery ratio and intensive treatment. Greater baseline ffERG compromise and the presence of SGF at 12 months were associated with poor functional recovery, underscoring the prognostic value of early retinal function assessments.

## Supplementary Information

Below is the link to the electronic supplementary material.

Supplementary file1 (DOCX 496 KB)

Supplementary file2 (DOCX 19 KB)

## Data Availability

No datasets were generated or analysed during the current study.
