# Supplementary material for: Full-field electroretinogram (ffERG) over 48 months correlates with baseline retinal dysfunction in Vogt-Koyanagi-Harada disease: a longitudinal study
Source: Doc Ophthalmol. 2026 Jan 30;152(2):185–96. doi: 10.1007/s10633-025-10080-9 (PMC13083520; doi:10.1007/s10633-025-10080-9)

**Supplementary figure**. Based on full-field electroretinogram parameters at 48 months, representative full-field electroretinogram (ffERG) waveforms and their corresponding amplitude values for each timepoint (M1, M6, M12, M48) from two patients with Vogt–Koyanagi–Harada disease are shown. (A) Patient with normal ffERG (Group 1) at 48 months. (B) Patient with subnormal ffERG (Group 2) at 48 months.


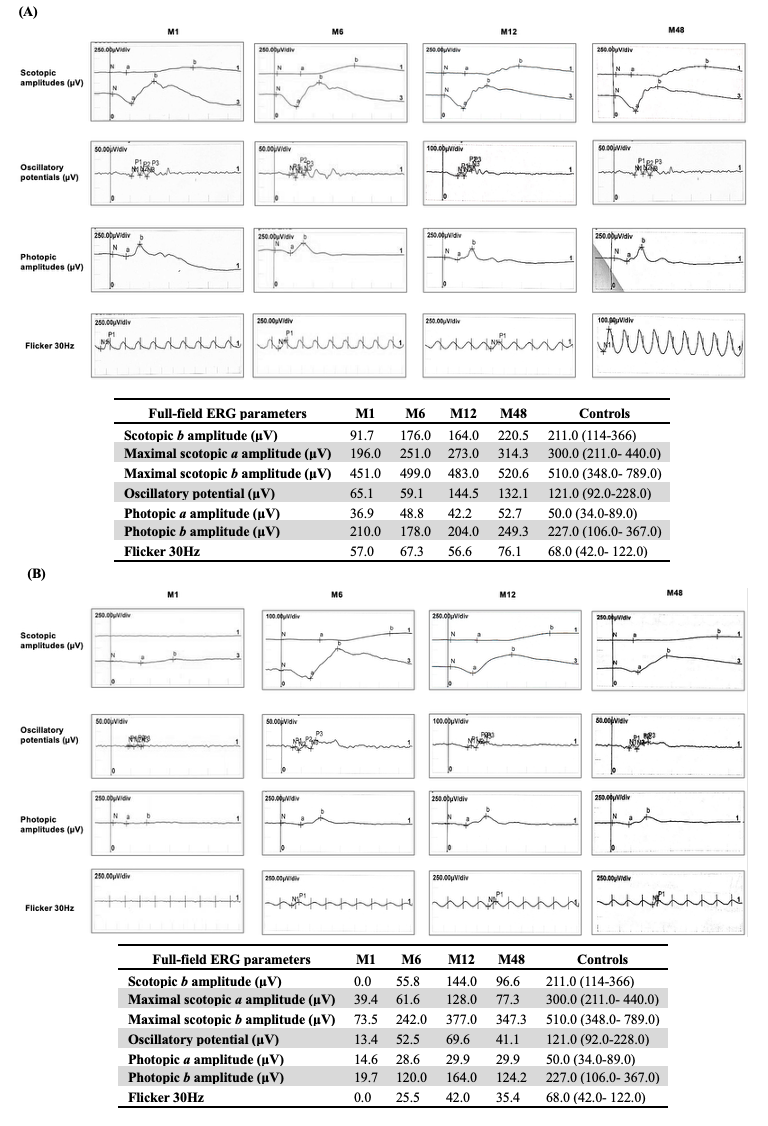

Supplement: Supplementary file 1 — Supplementary file1 (DOCX 496 KB) [file 10633_2025_10080_MOESM1_ESM.docx]
