# Supplementary material for: Full-field electroretinogram (ffERG) over 48 months correlates with baseline retinal dysfunction in Vogt-Koyanagi-Harada disease: a longitudinal study
Source: Doc Ophthalmol. 2026 Jan 30;152(2):185–96. doi: 10.1007/s10633-025-10080-9 (PMC13083520; doi:10.1007/s10633-025-10080-9)
Supplement: Supplementary file 2 — Supplementary file2 (DOCX 19 KB) [file 10633_2025_10080_MOESM2_ESM.docx]

**Supplementary table** Statistical analysis of comparisons between timepoints relative to M1 within each group and comparing of each timepoint between Groups 1 and 2.

| **Parameters** | **Groups** | **P** |
| --- | --- | --- |
| **Scotopic *b* amplitude (μV)** | **Group 1 vs Group 2 *** | **p<0.001** |
|  | M1 vs M6 | **p<0.001** |
|  | M1 vs M12 | **p<0.001** |
|  | M1 vs M48 | **p<0.001** |
| **Maximal scotopic *a* amplitude (μV)** | **Group 1** |  |
|  | M1 vs M6 | **p<0.001** |
|  | M1 vs M12 | **p=0.001** |
|  | M1 vs M48 | **p<0.001** |
|  | **Group 2** |  |
|  | M1 vs M6 | **p<0.001** |
|  | M1 vs M12 | **p=0.002** |
|  | M1 vs M48 | p=0.066 |
|  | **Group 1 vs Group 2** |  |
|  | M1-G1 vs M1-G2 | **p=0.03** |
|  | M6-G1 vs M6-G2 | **p=0.001** |
|  | M12-G1 vs M12-G2 | **p<0.001** |
|  | M48-G1 vs M48-G2 | **p<0.001** |
| **Maximal scotopic *b* amplitude (μV)** | **Group 1 vs Group 2 *** | **p<0.001** |
|  | M1 vs M6 | **p<0.001** |
|  | M1 vs M12 | **p<0.001** |
|  | M1 vs M48 | **p<0.001** |
| **Oscillatory potential (μV)** | **Group 1 vs Group 2 *** | **p<0.001** |
|  | M1 vs M6 | **p<0.001** |
|  | M1 vs M12 | **p<0.001** |
|  | M1 vs M48 | **p<0.001** |
| **Photopic *a* amplitude (μV)** | **Group 1 vs Group 2 *** | **P=0.01** |
| **Photopic *b* amplitude (μV)** | **Group 1** |  |
|  | M1 vs M6 | **p<0.001** |
|  | M1 vs M12 | **p<0.001** |
|  | M1 vs M48 | **p<0.001** |
|  | **Group 2** |  |
|  | M1 vs M6 | **p<0.001** |
|  | M1 vs M12 | **p<0.001** |
|  | M1 vs M48 | **p=0.002** |
|  | **Group 1 vs Group 2** |  |
|  | M1-G1 vs M1-G2 | p=0.81 |
|  | M6-G1 vs M6-G2 | p=0.863 |
|  | M12-G1 vs M12-G2 | p=0.051 |
|  | M48-G1 vs M48-G2 | **p<0.001** |
| **Flicker 30Hz** | **Group 1 vs Group 2 *** | **p=0.005** |
|  | M1 vs M6 | **p<0.001** |
|  | M1 vs M12 | **p<0.001** |
|  | M1 vs M48 | **p<0.001** |

*Similar behavior in the two groups along the evaluated moments

Generalized estimated equations with normal distribution and identity link function, supposing a correlation matrix AR-1 between moments and eyes: p-value < 0.05 indicated a statistical significance.
